# Tuning Insect Odorant Receptors

**DOI:** 10.3389/fncel.2018.00094

**Published:** 2018-04-05

**Authors:** Dieter Wicher

**Affiliations:** Department of Evolutionary Neuroethology, Max Planck Institute for Chemical Ecology (MPG), Jena, Germany

**Keywords:** chemoreception, olfaction, ionotropic receptor, odorant receptor, receptor kinase, GPCR, intracellular signaling

## Abstract

Among the insect olfactory receptors the odorant receptors (ORs) evolved in parallel to the onset of insect flight. A special property of this receptor type is the capability to adjust sensitivity of odor detection according to previous odor contacts. This article presents a current view on regulatory processes affecting the performance of ORs and proposes a model of mechanisms contributing to OR sensitization.

## Introduction

The performance of membrane proteins such as ion channels or receptors is dynamically adjusted according to changing physiological requirements. Olfactory receptors have to detect odors in a wide range of concentrations, from faint filaments at larger distance from the source to high concentrated and permanent presence near the source. In mammals, the olfactory receptors for general odors are G protein coupled receptors (GPCRs; Buck and Axel, 1991). For a comparison of vertebrate and insect olfaction see Kaupp (2010), for a recent review of insect olfactory receptors see Fleischer et al. (2018). Three types of receptor proteins detect volatile chemical information in insects. These are odorant receptors (ORs) which are restricted to insects, specific gustatory receptors (GRs) detecting carbon dioxide and receptors related to ionotropic glutamate receptors, called ionotropic receptors (IRs). The ORs evolved in parallel with the onset of insect flight (Missbach et al., 2014). Similar to GPCRs, insect ORs belong to the class of heptahelical transmembrane proteins. But compared with them, the OR proteins show an inverted orientation within the plasma membrane (Benton et al., 2006; Lundin et al., 2007; Smart et al., 2008). Analyzing the variation of insect OR protein amino acids during evolution revealed a model for transmembrane domain arrangement that is unrelated to GPCRs (Hopf et al., 2015).

An insect OR is a heteromeric construct formed by an odor-specific OrX protein and an ubiquitary odorant co-receptor, Orco (Larsson et al., 2004; Neuhaus et al., 2005). Heterologous coexpression of OrX and Orco proteins may in addition to the formation of ORs also lead to Orco homomers (German et al., 2013). It remains to be shown that the ciliar OSN membrane also comprises both types of constructs. At least for the soma membrane the insertion of Orco but not of Or22a/b proteins was demonstrated (Benton et al., 2006).

Experiments in heterologous expression systems supported the view that insect ORs primarily operate as ligand-gated channels (Sato et al., 2008; Wicher et al., 2008). An odor stimulation of sufficient strength produced—independent of G protein activity—an immediate transient response. The ORs form non-selective cation channels which are also permeable for Ca^2+^. At least some of these OR channels are constitutively active as their expression leads to an elevated level of free Ca^2+^, even in the absence of a stimulating odor. In addition to the fast ionotropic response there was a slowly developing OR current which relied on G protein function (Wicher et al., 2008). This finding raised the question whether there is also a role of metabotropic signaling in insect olfaction.

Intriguingly, when only Orco proteins are expressed they also form nonselective, Ca^2+^ permeable cation channels. These channels cannot be activated by odors but by cyclic nucleotides (Wicher et al., 2008). As in the case of ORs, it is presently unknown how the channels are composed of, either as dimers as the heptahelical channel rhodopsin (Müller et al., 2011; Kato et al., 2012), or as tetramer like conventional ion channels (Doyle et al., 1998). Orco dimer constructs have properties comparable to those of channels formed by native Orco proteins (Mukunda et al., 2014a).

Orco was found to be necessary for the insertion of the odor-specific receptor proteins into the plasma membrane (Larsson et al., 2004). An important signaling system during development is the hedgehog (Hh) system (Briscoe and Thérond, 2013). The transport of *Drosophila* ORs to and within the dendritic cilia is regulated by the Hh pathway (Sanchez et al., 2016). The localization of the ORs depends on the distribution of the Hh signal transducer Smoothened (Smo). Smo knockdown flies showed reduced odor responses indicating less expression while mutants in the Smo repressor Patched (Ptc) display largely enhanced odor responses. As Hh is produced in the OSNs the tuning of OR distribution is an autoregulatory process (Sanchez et al., 2016). Orco proteins possess a putative calmodulin (CaM) binding domain that is well conserved among insect species (Mukunda et al., 2014b). Robust mutations within this region of Orco proteins disrupted the OR traffic to the ciliar membrane (Bahk and Jones, 2016).

## G Proteins

That insect ORs—in spite of their inverted membrane topology—can interact with G proteins has been demonstrated for heterologously expressed ORs. Activation of *Drosophila* Or43a receptor could be monitored when it was coexpressed with the promiscuous G protein α subunit G_15_ in *Xenopus* ooytes (Wetzel et al., 2001). In addition, pheromone-induced activation of the silkmoth *Bombyx mori* OR-1 and 3 (Grosse-Wilde et al., 2006) and *Heliothis virescens* HR13 (Grosse-Wilde et al., 2007) coexpressed with G_15_ in T-Rex293 cells was reflected by calcium signals upon activation of IP_3_ receptors upon PLC activation via G_15_.

In the antenna of *Drosophila* all subunits of heterotrimeric G proteins were shown to be expressed (Boto et al., 2010). According to immunohistochemical studies, G_s_, G_i_ and G_q_ α subunits could be detected in the OSNs. This also includes the sensilla along which G_i_ and G_q_ were found, whereas G_s_ staining was seen at the basal segment (Boto et al., 2010). Expression of G_s_ in fly sensilla was also reported, and G_s_ proteins were found to be important for sensitive odor detection (Deng et al., 2011). In the antenna of *B. mori* the three α subunits G_s_, G_i_ and G_q_ were detected (Miura et al., 2005) while in the mosquito *Anopheles* females only G_q_ was found in certain sensilla (Rützler et al., 2006).

A role of G_o_ in *Drosophila* olfactory reception was shown by expression of the inhibitor pertussis toxin (PTX). Electroantennogram responses and the rise in spike frequency upon odor stimulation were reduced when PTX was expressed in the OSNs (Chatterjee et al., 2009). An effect of G_o/i_ inhibition by PTX was also observed for heterologously expressed ORs (Or22a plus Orco; Ignatious Raja et al., 2014). Monitoring calcium responses in Or22a expressing OSNs to odor stimulation in intact *Drosophila* antenna revealed weaker responses when G_o/i_ were inhibited (Ignatious Raja et al., 2014). Also the involvement of G_s_ proteins in OR signal transduction was reported (Deng et al., 2011). In addition, other studies demonstrated the importance of G_q_ proteins (Kain et al., 2008, 2009). Mutations in *dgq*, the gene encoding the *Drosophila* G_q_ α subunit caused reduced responses to odor stimulation.

In contrast to these findings, only tiny effects in *Drosophila* sensillum recordings were observed when manipulating the activity of G proteins (Yao and Carlson, 2010). Rather mild effects of G protein inhibition on Ca^2+^ responses were seen in heterologously expressed ORs (Smart et al., 2008).

## Second Messenger Systems

Independent of G protein-coupled signal cascades Ca^2+^ is an ubiquitous messenger that regulates the activity of proteins and links such signaling cascades. OR activation leads to Ca^2+^ influx into OSNs. Prolonged odor stimuli lead to a Ca^2+^-induced adaptation of the odor response (Cao et al., 2016). On the other hand, CaM activity can enhance the OR response to moderate stimuli (Mukunda et al., 2014b).

Mutations in the cascade downstream G_s_, i.e., in the adenylyl cyclase *rutabaga* and in the phosphodiesterase *dunce* affected the olfaction-guided behavior (Martín et al., 2001). Especially overexpression of *dunce* in specific OSNs which diminished the cAMP level in these cells produced severe phenotypes (Gomez-Diaz et al., 2004). A reduced cAMP level impairs the ability of flies to correctly detect an odor (Murmu and Martin, 2016). On the other hand, odor stimulation leads to enhanced cAMP production (Miazzi et al., 2016). That this effect was related to ORs had been suggested by the finding that odor stimulation of ORs expressed in HEK293 cells gave rise to enhanced cAMP production (Wicher et al., 2008). Artificially enhancing the cAMP concentration in *Drosophila* OSNs by injecting the membrane-permeable 8-bromo-cAMP or the adenylyl cyclase activator forskolin into the base of sensilla enhanced the odor-response and shifted the concentration-dependence towards lower odor concentration (Getahun et al., 2013). Similarly, in flies expressing a light-activated adenylyl cyclase in OSNs the spike activity could be enhanced by light exposure (Deng et al., 2011).

The signaling cascade downstream G_q_ also plays a role in odorant signal processing of insects (Krieger and Breer, 1999; Kain et al., 2008). In the hawkmoth *Manduca sexta*, pheromone stimuli are detected via PLC-dependent signaling (review, Stengl, 2010). Short and faint pheromone presentation causes an immediate increase spike activity in the receptor neuron which is accompanied by a transient rise in IP_3_ (Breer et al., 1990; Boekhoff et al., 1993). In cultured receptor neurons, IP_3_ perfusion opened a Ca^2+^ channel, the Ca^2+^ rise in turn activated further types of ion channels (Stengl et al., 1992; Stengl, 1993, 1994). While the pheromone signal transduction in *Manduca* seems to employ solely metabotropic mechanisms (Nolte et al., 2013, 2016), heterologously expressed pheromone receptors of the silkmoth *Bombyx mori* were found to act as ligand-gated channels (Sato et al., 2008). This indicates that pheromone signals might be processed via ionotropic and/or metabotropic mechanisms.

In *Drosophila*, *norpA* mutants that express a PLC enzyme with impaired function show reduced odorant responses (Riesgo-Escovar et al., 1995). An attenuation of odor responses was also observed in *plc21* mutants which express another defective PLC protein (Kain et al., 2008). Mutants in *stmbhA*, a gene encoding a putative PIP_2_-DAG lipase, show a markedly reduced electroantennogram response to odor stimulation (Kain et al., 2009). Thus, a disturbed PIP_2_ cleavage and regeneration cycle negatively affects odor information processing in insect OSNs.

Ca^2+^ signaling is employed by various pathways necessary for appropriate odor perception. One aspect of the G_q_ signaling cascade are Ca^2+^ signals produced when the PIP_2_ cleavage product IP_3_ activates IP_3_ receptors (IP_3_Rs) which release Ca^2+^ from the endoplasmic reticulum. In mutants with disrupted RyR and IP_3_R signaling the adaptation to odor signals is impaired (Murmu et al., 2011; Murmu and Martin, 2016). A role of intracellular stores for odor signal amplification was also observed *in vitro* (Ignatious Raja et al., 2014). In mammals, a broad dynamic range of the OSNs in terms of odor concentration relies on a proper function of mitochondria (Fluegge et al., 2012).

There is also a crosstalk between the G_s_ signaling cascade and intracellular Ca^2+^ signaling. Given that Orco proteins form cyclic nucleotide-activated ion channels permeable to Ca^2+^ (Wicher et al., 2008), an enhanced cAMP production may stimulate a Ca^2+^ influx into the OSNs.

Depending on the situation alternative messenger systems may be recruited while others are switched off. In *Manduca* pheromone receptor neurons, strong stimuli activate receptor guanylyl cyclases which lead to prolonged adaptation of neuronal activity. Furthermore, in the activity state the basal cAMP level is elevated, e.g., by octopaminergic signaling (Flecke et al., 2010), whereas the cGMP level is low, while at rest the cGMP level rises and the cAMP level drops (review see Stengl, 2010).

Second messenger signaling usually takes place within the sensory neuron. For silkmoth sex pheromone receptors an extracellular modulation has been observed (Nakagawa and Touhara, 2014). Extracellularly presented cyclic nucleotides were seen to weakly activate the *Bombyx* Or1/Orco complex and to inhibit the response to the sex pheromone bombykol.

## Sensitization of OR Response

**Figure 1:**
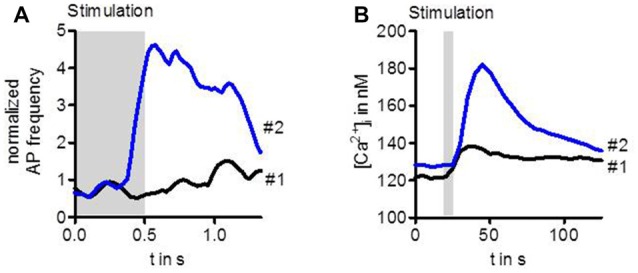
Sensitization observed in the activity of *Drosophila* OSNs **(A)** and in Ca^2+^ level of cultured cells expressing odorant receptors (ORs) **(B)**. The first weak odor stimulation did not enhance the spike frequency in OSNs expressing Or22a. However, the same stimulus repeated 20 s later elicited a robust increase in OSN activity **(A)**. Similarly, a first stimulation of HEK293 cells expressing Or22a + Orco enhanced the intracellular Ca^2+^ concentration [Ca^2+^]_i_ only slightly whereas the second stimulus led to a strong rise **(B)**. For experimental information see Getahun et al. (2013) **(A)** and Mukunda et al. (2016) **(B)**.

Stimulation of ORs with highly diluted odor below the detection threshold does not enhance the activity of the OSN (Figure 1A). However, when after a couple of seconds the same stimulus is presented again, the OSN can now respond with transiently enhanced spike frequency (Getahun et al., 2013). Similarly, an enhanced response after repeated gentle stimulation also leads to a rise in the intracellular Ca^2+^ concentration (Figure 1B, Mukunda et al., 2016). Thus, there must be an up-regulation of OR sensitivity during the interval between these stimuli. This sensitization could be mimicked by upregulation of cAMP production with the adenylyl cyclase activator forskolin. On the other hand, the OR sensitization can be suppressed by inhibition of cAMP production (Getahun et al., 2013). Another way to mimick sensitization is to activate protein kinase C (PKC; Getahun et al., 2013).

Thus a main player in the sensitization process seems to be a protein affected by cAMP and PKC. A known target for cAMP and PKC is Orco. Heterologously expressed Orco proteins form ion channels activated by cyclic nucleotides (Wicher et al., 2008). Orco activation by cAMP requires a certain level of phosphorylation by PKC (Sargsyan et al., 2011). The Orco PKC site S289 was seen to be specifically important for OR sensitivity (Guo et al., 2017).

With strong intracellular Ca^2+^ buffering that inactivates PKC, no Orco activation by cyclic nucleotides could be observed (Sato et al., 2008; Jones et al., 2011). On the other hand, PKC phosphorylation can activate Orco even in the absence of cAMP (Sargsyan et al., 2011). An Orco mutant that cannot be phosphorylated by PKC is insensitive to cAMP, i.e., the ion channel formed by Orco cannot be activated by cAMP (Sargsyan et al., 2011). In flies expressing this modified Orco protein the OR sensitivity is not enhanced by repeated odor stimulation at subthreshold concentration (Getahun et al., 2013). Also a forskolin-induced stimulation of cAMP production did not enhance the odor response as it was observed in wt flies.

When a rise in the cAMP level may sensitize ORs, the question arises whether an odor stimulus could initiate cAMP production. Using flies in which the OR-expressing OSNs coexpress a cAMP reporter, it was found that indeed odor stimulation caused an increase in cAMP concentration (Miazzi et al., 2016). Interestingly, in OSNs that lack an odor-specific OR protein but express Orco, odor stimuli did not change the cAMP level but Orco activation by the synthetic agonist VUAA1 let to a rise in cAMP. This might be due to activation of a Ca^2+^-dependent adenylyl cyclase as depolarization had the same effect (Miazzi et al., 2016).

**Figure 2:**
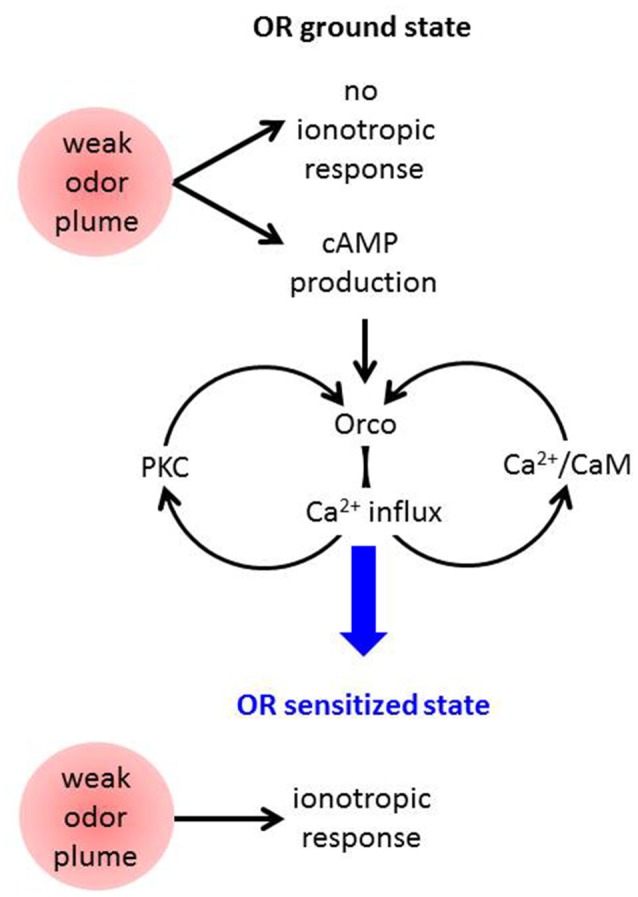
Schematic view on mechanisms assumed to contribute to *Drosophila* OR sensitization. A weak odor plume does not elicit an ionotropic response when ORs are in the basal state but it leads to enhanced cAMP production. This activates Orco channels causing Ca^2+^ influx into the OSN. Ca^2+^ may activate protein kinase C (PKC) and CaM, both proteins act on Orco and lead to stronger Ca^2+^ influx, i.e., there are two feedback loops. Finally the ORs become sensitized and are capable of responding to another weak odor plume with an ionotropic response which excites the receptor neuron.

These results are compatible with the following model of OR sensitization (Figure 2). An odor stimulus too weak to robustly activate the OR channel leads to OrX-dependent and/or Ca^2+^-dependent cAMP production (Miazzi et al., 2016). cAMP in turn activates Orco which causes a cation influx including Ca^2+^ import. This may activate two feedback loops. First, Ca^2+^-activated calmodulin (CaM) can bind to Orco and enhance the Ca^2+^ influx (Mukunda et al., 2014b). The requirement of CaM function for OR sensitization has already been shown (Mukunda et al., 2016). And second, Ca^2+^ may activate PKC enzymes to phosphorylate Orco which also enhances the ion flow through these channels (Sargsyan et al., 2011). Taken together, the parallel signaling loops via PKC and CaM initiated by cAMP-induced Ca^2+^ influx through Orco both amplify the Ca^2+^ influx further until the ORs are tuned to the deserved sensitivity. In terms of this model also other sources of intracellular Ca^2+^ signals, e.g., from intracellular stores might initiate these loops. Even Orco may provide such signal as it was seen to show constitutive activity (Wicher et al., 2008).

In addition to improve the performance of Orco, CaM can also modify the function of the OR constructs which depends on the odor-specific OrX protein (Mukunda et al., 2014b). In this study it was, for example, observed that CaM markedly prolonged the current through the *Drosophila* geosmin receptor Or56a/Orco that detects the presence of harmful microbes (Stensmyr et al., 2012).

Among the insect olfactory receptors the ability to become sensitized by repetitive stimulation is restricted to ORs and was not observed with IRs (Getahun et al., 2013). The equipment of flying insects with tunable ORs might have qualified these animals to detect faint odor plumes during flight (Getahun et al., 2016). There are certainly many more mechanisms that contribute to receptor sensitization such as an enhanced OR expression level at a circadian time when flies are highly sensitive to odor cues (Tanoue et al., 2008).

## Desensitization and Adaptation of OR Response

To appropriately process strong and/or maintained odor stimuli the insect olfactory system has to be able to downregulate the response in use-dependent manner. Long lasting stimulation and repetitive stimulation of sufficient strength leads to an adaptation of the OR response which is described by the Weber-Fechner relation (Nagel and Wilson, 2011; Cao et al., 2016). Under these conditions, the odor response becomes reduced and delayed. The Ca^2+^ influx during stimuli orchestrates the adaptive regulation of odor response (Cao et al., 2016).

One mechanism contributing to adaptation, a downregulation of Orco expression, was observed at elevated temperatures which cause enhanced odor concentration in the gas phase (Riveron et al., 2013) or upon excessive ethanol exposure (Morozova et al., 2006). Another way to reduce the OR sensitivity is Orco dephosphorylation at S289, as observed for prolonged odor exposure (Guo et al., 2017).

An adapting response also allowed to perceive turbulent odor filaments (Gorur-Shandilya et al., 2017). The processing of such stimuli is performed in two steps, first in the adaptation to the average odor strength which delays the response, and second in accelerating the onset of spiking. This in conjunction allows the correctly timed perception of odor plumes independent of their intensity (Gorur-Shandilya et al., 2017; Jacob et al., 2017).

## Orco Channel: Pacemaker or Regulator?

A role of Orco as pacemaker channel controlling the activity of OSNs was suggested recently (Stengl and Funk, 2013). Depolarizing ion channels opening in the range of the resting membrane potential are capable of shifting the membrane potential towards the threshold for action potential generation. As Orco proteins form cation channels activated by cyclic nucleotides and/or phosphorylation by PKC, its activation depolarizes the OSN membrane and thus should act as pacemaker (Stengl, 2010; Stengl and Funk, 2013). For *Manduca* pheromone receptors such a role is compatible with experimental findings (Nolte et al., 2013, 2016).

In *Drosophila* OSNs, the background activity is determined by the type of expressed OrX receptor protein (Hallem et al., 2004). The Δhalo mutant, an ab3A neuron lacking Or22a (Dobritsa et al., 2003), the spontaneous firing rate is very low which indicates a weak or missing pacemaker role of Orco (Hallem et al., 2004). Expression of OrX proteins led to a considerably enhanced spiking. The spike frequency varied between a few Hz for Or59b or Or22a and >60 Hz for Or47b (Hallem et al., 2004). Orco stimulation in *Drosophila* ab3A neurons with cAMP did not enhance their spontaneous activity (Getahun et al., 2013). However, odor stimulation of Or22a gave rise to a pacemaker activity and accelerates OSN spiking. A strong stimulation of OSN activity was also observed by administration of the synthetic OR agonist VUAA1 (Getahun et al., 2013). Although VUAA1 is capable of activating Orco, it is more efficiently in activation ORs (Jones et al., 2011). These observations support the above notion that in *Drosophila* OSNs OR activation but not Orco activation produces a pacemaker activity.

The missing pacemaker role of Orco in *Drosophila* OSNs is surprising insofar as heterologously expressed Orco proteins form spontaneously active channels (Sargsyan et al., 2011). And such leaky channels are known to lead to oscillations of the resting membrane potential which facilitates the triggering of action potentials (Stengl, 2010). Probably the number of Orco channels in the ciliar membrane might be too low to provide an efficient pacemaker conductance. By contrast, the Ca^2+^ influx into the receptor neurons activated by Orco activation would be sufficient to act as intracellular messenger. By this means, Ca^2+^-dependent proteins such as PLC, PKC or CaM could be activated, thereby facilitating OR sensitization.

## Author Contributions

DW wrote the manuscript.

## Conflict of Interest Statement

The author declares that the research was conducted in the absence of any commercial or financial relationships that could be construed as a potential conflict of interest. The handling editor is currently co-organizing a Research Topic with the author DW, and confirms the absence of any other collaboration.
